# Starch nanoparticles prepared by enzymatic hydrolysis and self-assembly of short-chain glucans

**DOI:** 10.1007/s10068-020-00768-w

**Published:** 2020-05-07

**Authors:** Seon-Min Oh, Byung-Hoo Lee, Dong-Ho Seo, Hyun-Wook Choi, Byung-Yong Kim, Moo-Yeol Baik

**Affiliations:** 1 Department of Food Science and Biotechnology, Institute of Life Science and Resources, Graduate School of Biotechnology, Kyung Hee University, Yongin, 17104 Republic of Korea; 2 Department of Food Science and Biotechnology, Gachon University, Seongnam, Republic of Korea; 3 Department of Food Science and Technology, Jeonbuk National University, Jeonju, Republic of Korea; 4 Department of Functional Food and Biotechnology, Jeonju University, Jeonju, Republic of Korea

**Keywords:** Starch nanoparticle, Enzymatic hydrolysis, Short-chain glucan, Physicochemical property

## Abstract

Enzymatic hydrolysis and self-assembly are considered promising methods for preparation of starch nanoparticles (SNPs) because they are environmentally friendly, and time- and cost-effective. These methods are based on the self-assembly of short-chain glucans released from the α-1,6 bonds in amylopectin. Since their discovery, many studies have described the structural and physicochemical properties of self-assembled SNPs. Self-assembled SNPs can be prepared by two methods: using only the soluble portion containing the short-chain glucans, or using the whole hydrolyzate including both insoluble and soluble fractions. Although the structural and physical properties of self-assembled SNPs can be attributed to the composition of the hydrolyzates that participate in self-assembly, this aspect has not yet been discussed. This review focuses on SNPs self-assembled with only soluble short-chain glucans and addresses their characteristics, including formation mechanisms as well as structural and physicochemical properties, compared with SNPs prepared with total hydrolyzates.

## Introduction

Since the emergence of nanotechnology, its use in the development and application of products has expanded in many industries. Today, nanoscience is applied in various fields such as packaging, electronics, energy production, pharmaceuticals, and the food industry (Khan et al., 2018). Nanotechnology is defined as the production of materials with a dimension between 1 and 1000 nm (Ahmadi and Jafarizadeh-Malmiri, 2020; Rao and Geckeler, 2011), and is of great research interest because of its unique characteristics that differ from bulk materials. The submicron size allows these materials to improve physical properties such as high surface-to-volume ratio, solubility, and dispensability (Jeong and Shin, 2018; Liu et al., 2009).

Nanoparticles in the food industry are classified according to if they are used as food additives (nano inside) or for food packaging (nano outside) (Samal, 2017). Nano inside is used to improve food quality in terms of shelf-life, texture and flavor. Nano outside is applied to increase shelf-life for safety, prevent gas flow or detect pathogens (Ravichandran, 2010). Duncan (2011) also described three ways to use nanoparticles: (1) to improve food quality, including texture and flavor in food processing, (2) to create an impermeable film to inhibit pathogens in food packaging, and (3) to improve and maintain bioavailability and stability of the nutrients in functional ingredients. Natural polymers are regarded as suitable and promising materials for preparation of nanoparticles due to being renewable, non-toxic and degradable (Li et al., 2016).

As a natural polymer, starch is non-toxic, biodegradable and inexpensive and is used widely in many industries other than the food industry. Starches are generally isolated from plant seeds, roots, tubers, stems and leaves, and their physicochemical properties depend on the plant origin and the ratio of amylose to amylopectin (Qiu et al., 2016; Singh et al., 2010). Starch nanoparticles (SNPs) have received much attention as sustainable and eco-friendly materials to help solve the depletion of fossil fuels and environmental pollution (Jiang et al., 2016c; Luo et al., 2019a). In addition, SNPs have potential as a precursor for various biomedical, pharmaceutical, and industrial applications, including implant material, plastic filler, and drug delivery carriers (Qiu et al., 2016; Yang et al., 2017). In general, there are two approaches in preparation of SNPs: top-down, where bulk material is broken down to obtain the nanoparticles, and bottom-up, through the build-up and aggregation of molecules (Tan et al., 2009). Top-down methods include ultra-sonication (Haaj et al., 2013; Jambrak et al., 2010), gamma irradiation (Lamanna et al., 2013; Yu and Wang, 2007), reactive extrusion (Song et al., 2011), high-pressure homogenization (Liu et al., 2009), acid hydrolysis (Kim et al., 2012; Putaux et al., 2003), emulsion-crosslinking (Ahmadi and Jafarizadeh-Malmiri, 2020; Chin et al., 2014),and enzyme treatment without gelatinization (Kim et al., 2008). Bottom-up methods include nanoprecipitation (Chin et al., 2011; Qin et al., 2016) and self-assembly of glucan chains produced by the debranching of isoamylase or pullulanase. The latter method is often referred to as ‘enzymatic hydrolysis and recrystallization’. Technically speaking, however, it seems inaccurate to call this method ‘recrystallization’ because this implies that an amorphous structure is transformed into an ordered structure by re-association between the starch molecules. ‘Recrystallization’ has a stronger meaning of molecular rearrangement during storage after parent starch is gelatinized. However, parent starch has been enzymatically hydrolyzed, and this changes the chain length and molecular mobility of short chain glucans. The SNP produced via this process have different crystal structure, relative crystallinity, digestibility, and thermal properties with the parent starch, suggesting that they are regarded as another new starch-based material. Therefore, the self-assembly and formation of the double helical structure between the short-chain glucans produced by the enzyme is more appropriately regarded as ‘crystallization’ rather than ‘recrystallization’, so this review will refer to it as ‘enzymatic hydrolysis and crystallization’ or ‘enzymatic hydrolysis and self-assembly’.

Enzymatic hydrolysis and crystallization was first attempted by Sun et al. (2014b), and SNPs of 20–100 nm were obtained from low molecular weight polymers produced by pullulanase hydrolysis. This method was an innovative approach (Le Corre and Angellier-Coussy, 2014; Suriya et al., 2018) in that it overcame several drawbacks of other methods such as low yield, high cost and environmental problems. It was also time-effective and easy to scale up. In addition, the resistant starch (RS) and slowly digestible starch (SDS) were increased, enhancing the health benefits (Liu et al., 2016).

**Fig. 1:**
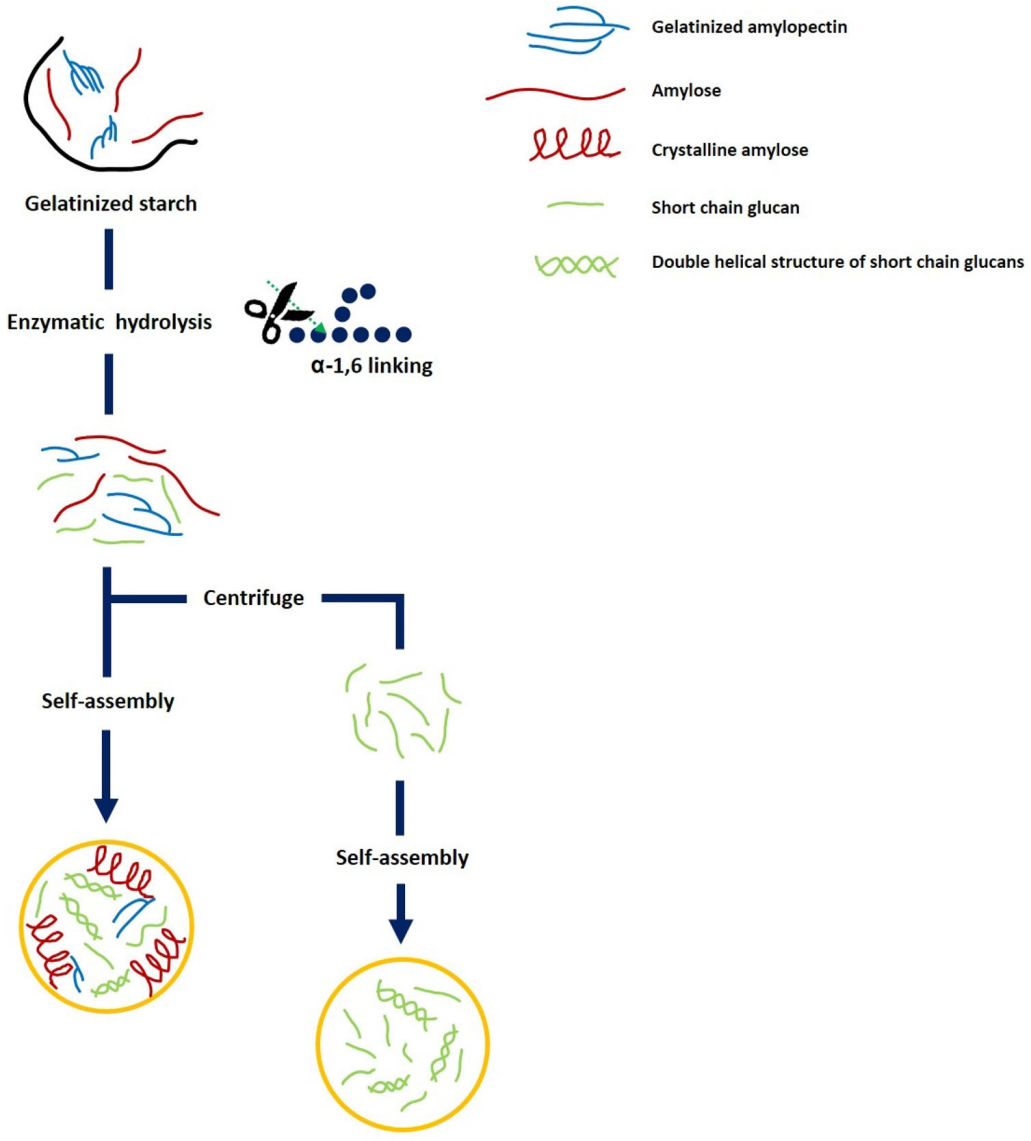
Schematic image of preparation of self-assembled SNPs

Properties of self-assembled SNPs differ according to the composition of the hydrolyzates in the self-assembly process (Fig. 1), which is divided into two cases: (1) using only the supernatant (soluble fraction) after enzymatic hydrolysis (Lee et al., 2019; Qiu et al., 2016; Sun et al., 2014a) and (2) using both the soluble and the insoluble fractions after enzymatic hydrolysis (Sun et al., 2014b; Suriya et al., 2018). In the former case, only the short-chain glucans of low molecular weight participate in the synthesis of SNPs (Table 1). However, the total hydrolyzate contains residues that are partially crystalline or branched polymers as well as linear glucans from hydrolysis. Although both cases fall into the category of ‘enzymatic hydrolysis and self-assembly’, they can be considered completely different SNPs at the micro- and molecular levels depending on the polymer composition (branched or unbranched, long or short chains). However, there has been no discussion on the composition of the hydrolyzate used in the SNP self-assembly process. This review focused and discussed on the formation and physicochemical properties of SNPs derived from only short-chain glucans with low molecular weight. This in-depth report of self-assembled SNPs will be helpful to identify and understand the differences between the composition of polymers used in self-assembly.

**Table 1:** SNPs prepared by self-assembly with only short-chain glucans

Starch	Self-assembly conditions	References
Waxy rice	Pullulanase hydrolysis at 58 °C for 8 h and discarding precipitate, followed by self-assembly at 4 °C for 8 h, annealing at 55 °C for 6, 12, 24, and 48 h	Ji et al. (2019)
Waxy maize	Pullulanase hydrolysis at 58 °C for 8 h and discarding precipitate, then addition of 0.1% of surfactant (SDS, Span 80 and Tween 80) based on solution, followed by self-assembly at 4 °C for 8 h Isoamylase hydrolysis at 40 °C for 24 and 48 h and discarding precipitate, followed by self-assembly at 4, 25 and 50 °C for 96 h Pullulanase hydrolysis at 58 °C for 8 h and discarding precipitate, followed by self-assembly at 4 °C for 8 h Isoamylase hydrolysis at 50 °C for 24 h and discarding precipitate, followed by self-assembly at 4, 25 and 50 °C for 24 h Pullulanase hydrolysis at 60 °C for 24 h and discarding precipitate, followed by self-assembly at 4 °C for 24 h Pullulanase hydrolysis at 37 °C for 24 h and discarding precipitate, and addition of chitosan to a final concentration of 0.05%, followed by self-assembly at 4 °C for 24 h	Li et al. (2016) Lee et al. (2019) Jiang et al. (2016b) Jiang et al. (2018) Ji et al. (2015) Cai and Shi (2014) Luo et al. (2019b) Luo et al. (2019a)
Waxy potato	Isoamylase hydrolysis at 50 °C for 24 h and discarding precipitate, followed by self-assembly at 50 °C for 24 h	Cai and Shi (2014)
Potato	Pullulanase hydrolysis at 58 °C for 8 h and discarding precipitate, followed by self-assembly at 4 °C for 8 h	Jiang et al. (2016c)
	Pullulanase hydrolysis at 58 °C for 8 h and discarding precipitate, then addition of 0.1% of surfactant (SDS, Span 80 and Tween 80) based on solution, followed by self-assembly at 4 °C for 8 h	Li et al. (2016)
Proso millet	Pullulanase hydrolysis at 56 °C for 8 h and discarding precipitate, followed by self-assembly at 4 °C for 0.5, 4, 12 and 24 h	Sun et al. (2014a) Gong et al. (2016)
Maize	Pullulanase hydrolysis at 60 °C for 24 h and discarding precipitate, followed by self-assembly at 4 °C for 24 h	Luo et al. (2019b)
	Pullulanase hydrolysis at 56 °C for 8 h and discarding precipitate, followed by self-assembly at 4 °C for 12 h	Liu et al. (2016)
Chestnut	Pullulanase hydrolysis at 60 °C for 24 h and discarding precipitate, followed by self-assembly at 4 °C for 24 h	Luo et al. (2019b)
Taro	Pullulanase hydrolysis at 58 °C for 8 h and discarding precipitate, then self-assembly at 4 °C for 8 h, followed by modification with octenyl succinic anhydride	Jiang et al. (2016a)

## Structure of starch

Starch consists of two components: amylose, which is generally a polymer consisting of α-1,4 glycosidic bonds and low content (less than 1.0%) of α-1,6 branch, and amylopectin, a branched polysaccharide composed of both α-1,4 and α-1,6 glycosidic bonds (Dufresne, 2014; Zobel, 1988). Starch granules form as semi-crystalline rings with alternating amorphous and crystalline clusters, or growth rings, between 100 and 1000 nm (Pérez and Bertoft, 2010; Wang et al., 2014). They are similar to the structure of an onion, with rings of approximately 9–10 nm. This periodicity is due to the crystalline and amorphous lamella found in the semi-crystalline shell. Recent studies have shown that semi-crystalline lamellas are densely packed into large, spherical blocklets with a diameter of 20–50 nm, depending on the plant source and its location in the granules (Baker et al., 2001; Gallant et al., 1997). The rate and extent of enzymatic hydrolysis of starch are affected by the arrangement of crystalline and amorphous regions in lamella or blocklets that form the starch granules (Blazek and Gilbert, 2010; Buléon et al., 1998; Gallant et al., 1997). Amylopectin branches can be classified into 3 types: A-, B- and C-chains. A-chains are unbranched polymers, whereas B-chains have one or more side chain and C-chains carry the sole reducing end group (Perin and Murano, 2017; Wang et al., 2014). In amylopectin, the mean degree of polymerization (DP) of short chains, including both inner and outer chains, ranges from 14 to 18, and that of long chains ranges from 45 to 55 (Buléon et al., 1998).

## Enzymatic hydrolysis and crystallization for SNPs

Preparation of SNPs has been attempted by hydrolysis of granular starch using α-amylase, but it is difficult to obtain nano-sized particles using this method and few studies used this enzyme solely (Kim et al., 2008; Li et al., 2017). Enzymatic hydrolysis of granular starch has been used mainly as a pretreatment of acid hydrolysis to produce SNPs, which is effective to reduce the production time by producing cracks and pores on the surface (Hao et al., 2018; Le Corre et al. 2011).

Recently, an innovative approach for enzymatic hydrolysis and crystallization for SNPs based on the debranching of amylopectin by debranching enzymes is provided. These debranching enzymes belong to a group of enzymes that participate in the hydrolysis of the α-1,6 glycoside bond of polyglucan (Nakamura, 1996). Branches with α-1,6-glycoside bonds in partial amylose and amylopectin can be broken down using debranching enzymes such as isoamylase and pullulanase (Manners, 1989). Isoamylase and pullulanase have different specific actions and the final chain structure depends on their hydrolytic behaviors (Hizukuri et al., 2006; Manners and Matheson, 1981). Pullulanase hydrolyzes exo-wise α-1,6 glycoside bonds of short branched chains such as amylopectin or pullulan and produces maltose or maltotriose. However, isoamylase acts as either an endo or exo enzyme in amylopectin and glycogen, respectively (Harada et al., 1972; Manners and Matheson, 1981).

In some studies, α-1,4 glucans produced by debranching are referred to as short-chain amylose or short-chain glucans (Cai et al., 2012; Cai and Shi, 2013; Luo et al., 2019a). These glucans released by amylopectin have a high mobility and can easily form a double helical structure via hydrogen bonds (Gong et al., 2016; Luo et al., 2019a). Although amylose chain with some α-1,6 glycosidic bonds are also debranched, the branches of amylose are generally very long and have a high molecular weight, which can be separated in the precipitation step and excluded from synthesizing SNPs. Thus, only final obtained short-chain glucans self-assemble and form double helix followed by crystallization. Self-assembly is a bottom-up approach which forms building block material composed of atoms, molecules, macromolecules and colloidal particles (Glotzer et al., 2004). It is regarded as a spontaneous phenomenon of molecules, resulting in a stable structure by non-covalent bonds under equilibrium conditions (Subramani et al., 2008). Pelesko (2007) classified molecular and nano scale into intra- and intermolecular self-assembly. In intramolecular self-assembly for SNP, the random coil arranges into stable and well-defined particles. In intermolecular self-assembly, assembled particles interact with surrounding particles, forming a supramolecular or quaternary structure. In the self-assembly process, morphology and geometry of the particles are affected by driving forces such as particle–particle and particle-environment (Jiang et al., 2011). Therefore, in preparation of SNP, randomly distributed short-chain glucans formed the double helical structure for stable state by the intermolecular self-assembly. While, at the same time, intramolecular interaction also occurs and produces amorphous or less-ordered structure in SNP.

Based on the rapid association of linear chains, Sun et al. (2014b) found that SNPs of 50–100 nm were prepared by pullulanase hydrolysis and self-assembly at 4 °C for 8 h. There are many reports of SNPs prepared by self-assembly of short-chain glucans in a variety of differing conditions such as enzyme concentration (Shi et al., 2018), time and temperature (Gong et al., 2016; Sun et al., 2014a; Suriya et al., 2018) and starch source (Luo et al., 2019a). In addition, the application and utilization of research on SNPs has expanded as physical and chemical modifications (Ji et al., 2015; 2019; Jiang et al., 2016a) and encapsulation (Ji et al., 2017; Lin et al., 2019) have been introduced. As mentioned previously, final products with different properties can be obtained depending on the hydrolyzates. These are grouped into resistant starch (Kiatponglarp et al., 2015; Ravichandran, 2010) in a broad sense, and are referred to variously as starch nanoparticles (Qiu et al., 2016; Sun et al., 2014a), nanostarch (Suriya et al., 2018) and short-chain amylose crystals (Cai and Shi, 2010; Zeng et al., 2016) in a narrower sense.

## Formation mechanism and yield

Starch retrogradation is another self-assembly process of typical starch molecules, resulting in an ordered structure through reassociation between starch molecules (Luo et al., 2019a). Thus, SNPs which form from short-chain glucans derived from starch have also been described as similar to those formed from starch retrogradation. SNPs formation takes place by two continuous steps: nucleation and crystal growth (Gong et al., 2016; Tian et al., 2012). The nucleation occurs quickly, followed by crystal growth progressed simultaneously. According to Sun et al. (2014b), linear glucans with a DP of 12–60 are involved in recrystallization, and these linear glucans form double helices, form clusters with hydrogen bonds, and then rearrange clusters. Meanwhile, Shi and Gao (2011) reported that formation of double helices requires short chain lengths with 20 < DP < 30. Buléon et al. (2007) reported that linear glucans form the double helical structures and these structures become ordered domains by different morphological orientations. Various parameters such as starch concentration, hydrolysis time, storage temperature, and time affect self-assembly formation (Cai and Shi, 2013; 2014; Lee et al., 2019; Sun et al., 2014a).

Sun et al. (2014a) reported that the yield of proso millet SNPs increased from 15.29 to 54.66% at 4 °C with increasing self-assembly time from 0.5 to 24 h, respectively. This means that association between the short-chain glucans is favored both low temperature and long time. Similarly, when glucans released by isoamylase hydrolysis for 24 or 48 h were stored at 4, 25 and 50 °C, the sample at 4 °C storage had the highest yield and the sample at 50 °C had the lowest yield (Lee et al., 2019). This result also indicates that low temperature storage is suitable for chain association, and relatively high temperature (50 °C) increases the mobility of molecules resulting in more propagation process than nucleation. However, self-assembly of short-chain glucans did not always match crystallization. According to Lee et al. (2019), SNPs obtained at 4 °C showed the highest yields, but SNPs at 25 °C had the highest relative crystallinity from XRD results. This is explained by the fact that fast chain association often forms the amorphous matrix and short chains are rather unfavorable for crystal formation. To further explore these interesting results, it is necessary to investigate the self-assembly and crystallization kinetics of SNPs.

In addition, the yield increased as the debranching time increased, because the amount of short-chain glucans available for SNP formation increased with increasing debranching time. Cai and Shi (2014) confirmed that samples with high starch concentration (25%), storage in low temperature (4 °C) and long chain length (average chain length, 32) had higher yields than other conditions. Luo et al. (2019b) found that initial materials are important in forming the SNPs. SNPs made from waxy maize starch showed discontinuous spherical shapes, while maize and chestnut starch, which have a higher amylose content (> 25%), had irregular and highly aggregated morphologies. This implies that amylose content affects the formation and morphology of SNPs. It could be considered that long-chain amylose could not be hydrolyzed by pullulanase but dissolved in aqueous solution generates a discontinuous shape by interfering self-assembly.

Highly agglomerated SNPs are limited in their industrial applications, and research into the production of monodisperse particles has been carried out. In order to produce discrete particles, slow propagation should be induced without additional nucleation once the nucleation occurs. Discontinuous particles can be produced under various interactions (van der waals, static electricity and attraction) in the self-assembly process. Luo et al. (2019a) found that addition of chitosan before self-assembly was effective for controlling nucleation and crystal growth with electrostatic repulsion at low pH and producing monodisperse particles. In addition, Li et al. (2016) designed nanoparticles with three surfactants (SDS, Span 80 and Tween 80) and controlled the formation and size of nanoparticles by preventing nucleus growth by electrostatic repulsion and steric hindrance. The formation of SNPs can be controlled by the surface activity of these compounds, which is an important consideration because it also affects the morphological properties of SNPs.

## Molecular weight distribution and degree of polymerization

Native starch has a wider molecular weight distribution than SNPs after hydrolysis because natural starch is composed of amylose and highly polymerized amylopectin. It has been reported that all self-assembled SNPs have bimodal molecular weight distribution regardless of the hydrolyzate composition (Cai and Shi, 2010; 2014; Lee et al., 2019; Luo et al., 2019b; Shi et al., 2018). According to Lee et al. (2019), the molecular weight distribution of SNPs is affected by the hydrolysis and crystallization time. In the case of isoamylase hydrolysis for 24 h, the ratio of fraction II which includes both the less debranched dextrin and the short chains, was higher, whereas hydrolysis for 48 h had a high ratio of fraction III, reflecting A-chains released from amylopectin. This is because further hydrolysis proceeds with time and the amount of short chains with low molecular weight increases.

Crystallization at higher temperatures also leads to the production of larger molecules than at lower temperatures, which favors long-chain glucan binding such as B-chains (Lee et al., 2019). On the contrary, Cai and Shi (2013) showed the same molecular weight distribution when they were crystallized at different temperatures using the same concentration, but found that the higher the starch concentration, the higher the low molecular weight ratio. This suggests that molecular weight distribution of SNPs is more dependent on debranching than crystallization. Also, added amount of enzyme is greatly influenced the molecular weight distribution of SNPs. When waxy corn SNPs are prepared with different enzyme concentrations, the percentage of low molecular weight SNPs increases, but there is no significant difference in DP (Luo et al., 2019b). On the other hand, compared with SNPs with total hydrolyzates, the peak area of low molecular fractions is quite different. Crystallized maize, waxy maize, potato, and pea starch showed the low molecular fractions of 66.77–77.36% (Liu et al., 2015), whereas SNPs with only short-chain glucans have higher low molecular fractions of over 90% (Luo et al., 2019b). This may be attributed to partial crystalline structures which are unbranched during hydrolysis and mixed into the SNPs.

Crystallized SNPs generally have many B1 (13 < DP < 24) chains. Waxy corn and potato SNPs have average DPs of 9.51 and 16.14, respectively (Li et al., 2016). Waxy corn SNPs obtained as a hydrolyzate with isoamylase showed more than 45% of DP 13–24 regardless of crystallization temperature, with an average DP of 22.9–25.3 (Lee et al., 2019). Shi et al. (2018) hydrolyzed the starch using various amounts of pullulanase and the DP significantly decreased as the amount of enzyme increased. However, the average DP was 14.6–20.4 which was included in the B1 chain. This agrees with the results of Liu et al. (2015). It was reported that a DP > 10 is required for formation of the double helical structure (Gidley and Bulpin, 1987), and Shi and Gao (2011) also suggested 20 < DP < 30 is a suitable chain length for crystallization and the formation of double strands. In addition, short-chain glucans with DP < 10 inhibit crystallization and remain in solution (Cai and Shi, 2010). Meanwhile, short-chain glucans of DP 20–35 are suitable to produce resistant starch (Cai and Shi, 2010; Park and Park, 2017; Shi et al., 2013), which is expected to have a significant effect on the digestibility of SNPs.

## Morphology

Many factors such as solid and enzyme concentration, self-assembly time and temperature, drying conditions and presence of surfactant affect the morphology and size of SNPs. Because the shape and size determine the structural and physicochemical properties in nanoparticles (Ku et al., 2014), investigating the morphology of SNPs is important and fundamental.

**Fig. 2:**
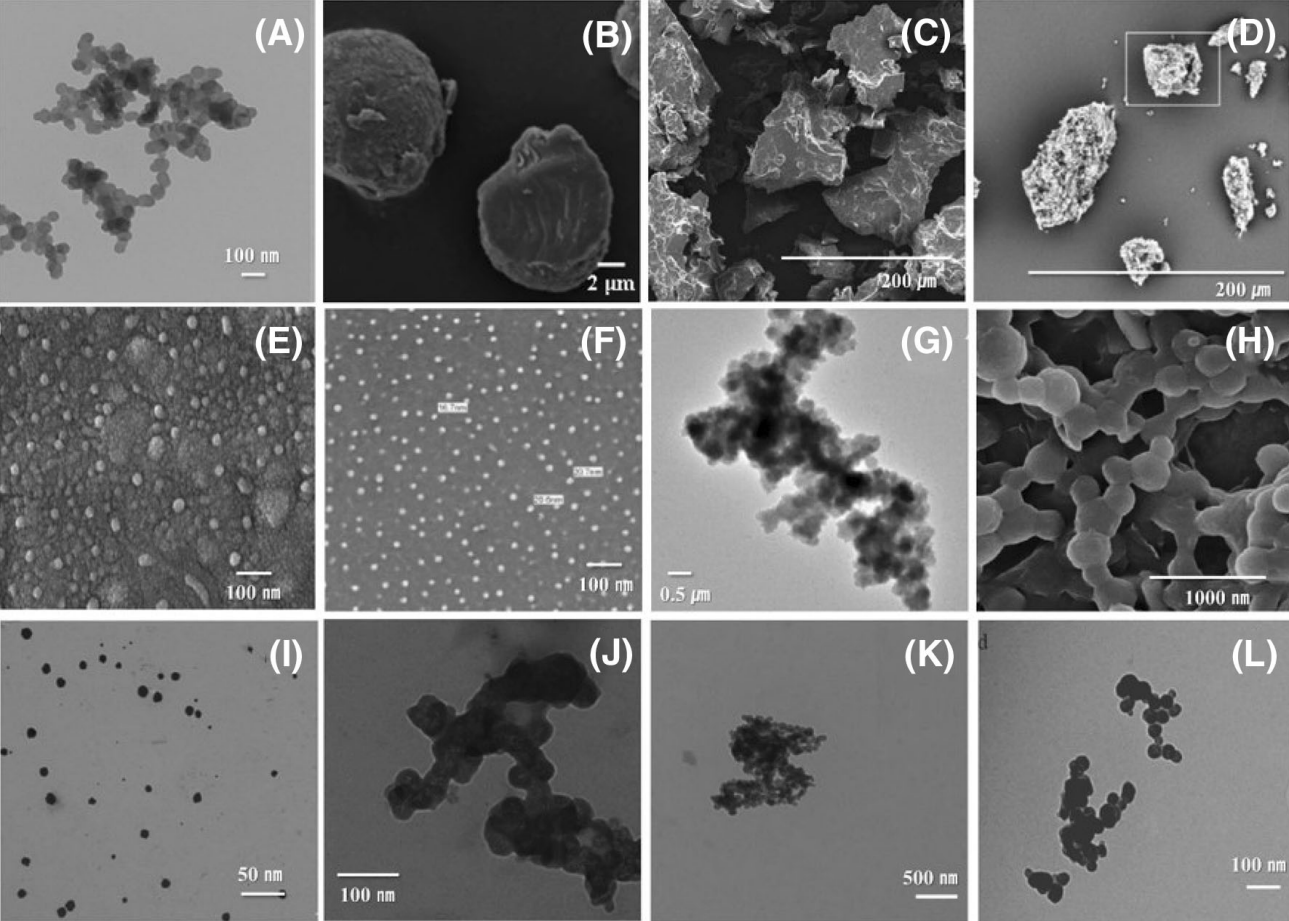
Morphological characteristics of SNPs depending on the hydrolysis composition; SNPs using total hydrolysate (**A**–**D**) and SNPs using short-chain glucans (**E**–**N**); **A** Sun et al. (2014b); **B** Cai and Shi (2013); **C** Miao et al. (2009); **D** Zeng et al. (2016); **E** Sun et al. (2014a); **F** Jiang et al. (2016c); **G** Lee et al. (2019); **H** Liu et al. (2016); **I** Li et al. (2016); **J** Jiang et al. (2018); **K** Jiang et al. (2016a; 2016b; 2016c); **L** Ji et al. (2019)

The morphological characteristics of SNPs obtained by self-assembly with only supernatant are compared with SNPs prepared by total hydrolyzates (Fig. 2). When self-assembled without discarding the sediment released by hydrolysis (Fig. 2A–D), the final products have varying morphology and some of them have smooth plate-like surface and irregular gel shape. Sun et al. (2014b) prepared SNPs by retrogradation of the total hydrolyzate of waxy corn starch at 4 °C for 8 h (Fig. 2A). The obtained SNPs were 50–120 nm and spherical, and it was found that the morphological characteristics were affected by the drying method rather than the starch concentration. SNPs from *Amorphophallus paeoniifolius* (elephant foot yam) starch produced in the same manner were reported to have an irregular or a spherical nanoparticle size of 18–26 nm (Suriya et al., 2018). Cai and Shi (2013) obtained micro- and smooth surface spherulites containing short-chain amylose debranched by isoamylase (Fig. 2B). In contrast, large and irregular shapes also have been reported (Demirkesen-Bicak et al., 2018; Miao et al., 2009; Shi et al., 2013; Zeng et al., 2016). When the residues and short-chain glucans from debranching were self-assembled together, a plate-like structure (Fig. 2C) or loose and porous structures (Fig. 2D) were confirmed, with a wide size distribution of 10–110 μm. These irregular final products can hardly be referred to as nano- or micro-particles but they contained high amount of slowly digestible starch and resistant starch. These morphological differences may be due to the degree of enzymatic hydrolysis, enzyme activity and attraction between molecules, however, the mechanism needs to be further investigated.

Meanwhile, self-assembly using supernatants containing only short-chain glucans results in formation of typical spheres (Fig. 2E–N). Sun et al. (2014a) prepared SNPs with different crystallization times (0.5–24 h) using only short-chain glucans of proso millet. The size of these SNPs ranged from 30 to 100 nm (Fig. 2E). Contrary to other studies, smaller nanoparticles were observed as the crystallization time increased. This interesting result may be due to rapid nucleation of shorter glucose units and rapid growth in an aqueous solution (Geng et al., 2012). Potato SNPs have a spherical shape of 15–30 nm (Fig. 2F), and relatively large particles (50–120 nm) are found when prepared in a 7.5% starch concentration. Using these ultra-sized nanoparticles, Jiang et al. (2016c) suggested their potential as a film material. Hydrolysis by isoamylase also produces aggregated round nanoparticles, and smaller particles are observed when crystallized at 50 °C instead of low temperatures (Lee et al., 2019) (Fig. 2G). The SNP of waxy corn starch obtained self-assembly were 200–300 nm which larger than starch nanocrystals (SNCs) of waxy corn starch obtained by H_2_SO_4_ hydrolysis (Fig. 2H) (Jiang et al., 2016b). The addition of additives during SNP synthesis also affects the morphology of the final SNPs. As a size-control technique, Li et al. (2016) added three surfactants (SDS, Span 80, and Tween 80) into the supernatant before self-assembly. The addition of surfactants is effective in reducing the size of the SNPs, and SNPs with Tween 80 had uniform and minimally aggregated spherical particles (Fig. 2I). In the presence of chitosan, smaller uniform-sized microparticles (~ 1 μm) were observed with a narrow size distribution (Fig. 2J) (Luo et al., 2019a). Recently, studies on chemically or physically modified SNPs have been reported to develop new modified SNPs (Fig. 2K, L). Jiang et al. (2016a) prepared OSA-SNPs with various degrees of substitution (DS) using taro SNPs and octenyl succinic anhydride (OSA), and observed that the particle size increased with increasing DS (Fig. 2K). Annealing of SNPs was carried out at 55 °C for 6, 12, and 24 h; however, there were no significant differences in morphology of the annealed SNPs (Fig. 2L) (Ji et al., 2015). SNPs with total hydrolyzate can be produced as spherical or irregular particles depending on various factors, whereas SNPs which self-assembled from only short-chain glucans have the advantage of relatively uniform size. This uniformity of particle size has better potential for applications such as drug delivery, nano-films and nano-fillers, although additional researches on controlling the size distribution and polydispersity is needed.

## Crystallinity

The crystal structure of native starch can be classified into A-, B- and C-types. A-type is mainly found in cereal starch, while B-type is found in tuber starch. C-type, a mixture of A- and B-type crystals, is observed in legume starch (Pérez and Bertoft, 2010; Wang et al., 2014). Depending on the preparation method and treatment, the crystal characteristics of SNPs may be identical or changed with that of native starch. The differences in these crystalline structures are attributed to the arrangement of the double helical structure and the relative amount of water bound in one-unit cell of crystal structure (Buléon et al., 1998; Pérez and Bertoft, 2010).

Osella et al. (2005) reported that retrograded starch has a B-type crystal pattern when the moisture content is more than 43%, whereas A-type crystals are obtained when the moisture content is less than 29%. In general, short-chain glucans after hydrolysis are present in the aqueous solution, and self-assembly and crystallization progress under high moisture condition. Thus, many studies which crystallized short-chain glucans in an aqueous solution at low temperature obtained B-type crystalline SNPs regardless of the starch source (Cai and Shi, 2010; Lee et al., 2019; Luo et al., 2019b; Sun et al., 2014a). Although native proso millet starch is a typical A-type starch, the proso millet SNPs prepared at 4 °C showed B-type crystals regardless of the crystallization time, and the relative crystallinity increased with longer storage time (Sun et al., 2014a). On the other hand, SNPs obtained from pullulanase hydrolysis followed by storage at 4 °C for 12 h revealed B-type crystal pattern, which differed from the crystalline properties of SNCs (A-type) prepared by acid hydrolysis (Liu et al., 2016).

B-type crystal SNPs are possibly due to not only the moisture content but also the DP of short-chain glucans released from amylopectin. It has been reported that A-type crystal was formed when the DP was in the range of 10–12, whereas B-type crystal was formed when DP was over 13 (Pfannemüller, 1987). Considering that the average DP of SNPs is generally 13 or more, the characteristic of the B-type crystal in the XRD corresponds to the DP of the SNPs.

Cai and Shi (2014) attempted to obtain A- and B-type crystal SNPs by designing crystallization environment in an aqueous solution. As a result, A- and B-type crystal patterns were mixed when the starch concentration was 15%, and A-type crystals were obtained when SNPs were prepared with a starch concentration of 25% and a crystallization temperature of 50 °C. Modulation of temperature and moisture content also showed a significant change in the crystal pattern of SNPs. Waxy corn SNPs crystallized at 4 and 25 °C showed typical B-type crystal pattern, and, A-type crystal pattern was observed when crystallized at 50 °C (Lee et al., 2019).

While, Ji et al. (2019) found that crystal pattern of B-type crystal SNPs did not change after annealing at 55 °C for 48 h. These results suggested that the effect of temperature in the crystallization step on the crystal pattern of SNPs is still unclear. However, longer annealing time causes higher crystallinity. This suggests that the structure inside the SNPs is getting more ordered during annealing. Unlike the annealing, heat moisture treatment (HMT) was effective in changing the crystalline structure of SNPs. When waxy corn SNPs with 30% moisture content were treated at 110 °C, the B-type crystal pattern changed to A-type, which signified that HMT altered the double helix movement and crystal orientation (Ji et al., 2015).

Meanwhile, the additives and chemical modification did not change the crystalline structure, but they changed the relative crystallinity of SNPs. The relative crystallinity decreased when SDS and Span 80 were added to waxy corn SNPs or potato SNPs, suggesting the inhibition of growth of SNPs and mobility of polysaccharides (Li et al., 2016). Jiang et al. (2016a) reported both OSA-SNPs and native SNPs revealed B-type crystal pattern because the modification occurs mainly in the amorphous region of SNPs. The relative crystallinity decreased as the degree of substitution increased, which is attributed to the alkali system using NaOH destroying the crystalline region when OSA reacts with the SNPs (Jiang et al., 2016a).

Compared with native starch, relative crystallinity of SNPs has been increased (Liu et al., 2016; Sun et al., 2014a; Suriya et al., 2018). This is because single helical polymers, especially short-chain glucans produced by debranching, tend to form double helices in aqueous systems, which are more involved in crystallization of SNPs (Sun et al., 2014a; 2014b). Crystallization of debranched starch molecules may cause different results because crystallization is a complex process involving chain alignment, morphological orientation and crystal packing, etc.

## Thermal properties

Characterization of the thermal behavior of SNPs was generally performed by differential scanning calorimetry (DSC). The thermal behavior of native starch and SNPs showed different results. Native proso millet starch melted at 76.74 °C and showed a relatively narrow endothermic peak with range of 12.20 °C. On the other hand, proso millet SNPs with different crystallization time revealed endothermic peak range of 44.21 °C. In addition, the melting enthalpy (ΔH) of native starch was 10.2 J/g and all SNPs showed lower ΔH, which can be explained by the decrease in both ordered structure and stable double helical structure during the self-assembly of short chain glucans (Sun et al., 2014a). These were also confirmed in thermogravimetric analysis (TGA) (Sun et al., 2014a; 2014b). The TGA curve can be divided into three regions; (1) initial weight loss due to evaporation of water, (2) the main decomposition of starch, and (3) the carbonization. The initial decomposition temperature of the native starch was 318 °C, and that of SNPs was much lower, within the range of 269–295 °C. Native starch has a higher onset decomposition temperature than SNP samples, indicating that the thermal stability of SNPs is reduced.

However, the thermal behavior of waxy maize SNPs revealed a different pattern. According to Luo et al. (2019b), the endothermic peak of waxy maize SNPs ranged from 69.9 to 104.72 °C, which is broader than native starch. Also, the ΔH of SNPs (17.41 J/g) was higher than that of native starch (8.41 J/g), indicating high proportion of the double helical structure via hydrogen bonds. Liu et al. (2016) compared the thermal properties of SNCs produced from acid hydrolysis and SNPs, and both nanoparticles had increased endothermic peak temperature and ΔH compared to native starch. These results are attributed to the hydrolysis of the amorphous region and strong rearrangement between starch molecules. Lee et al. (2019) reported that the thermal properties of SNPs are dependent on degree of debranching and crystallization time. With the same crystallization temperature, the endothermic peak range and ΔH increased as debranching times increased. When short chain glucans are produced during debranching, more thermally stable recrystallization proceeds and consequently imparts thermal stability to them. On the other hand, the ΔH of SNPs crystallized at 25 °C was higher than those crystallized at 4 and 50 °C, which was inconsistent with the yields. This result indicates that the association of short chain glucans is promoted at low temperature, but does not always lead to crystal formation (Lee et al., 2019).

Physical treatments such as annealing and HMT can contribute to the improvement of the thermal stability of SNPs. The endothermic peak of annealed SNPs shifts to higher temperature and improved thermal stability (Ji et al., 2019). The endothermic peak range of SNPs was 30.8 °C, but after annealing for 6 and 48 h, it narrowed to 27.4 and 25.3 °C, respectively. This may be due to the more perfect crystal structure of the annealed SNPs. HMT-SNPs also increased To, Tp, Tc and ΔH (Ji et al., 2015) possibly due to the formation of intermolecular hydrogen bonds during HMT. The increases in To, Tp and Tc were due to the formation of stable compositions formed as a result of the reorientation and crystallization of starch molecules (Sun et al., 2014b). Meanwhile, the thermal stability of chemically modified SNPs was weaker than that of native SNPs. Decomposition and weight loss of OSA-SNPs occurs at lower temperatures than those of native SNPs. Moreover, this was enhanced with increasing degree of substitution (Jiang et al., 2016a). This may be due to OSA denaturation, a partial disruption of the crystal structure of the SNPs, and a weakening of the interaction between short-chain glucans with the introduction of hydrophobic groups of OSA (Bao et al., 2003).

Depending on the type of additives, the thermal stability of the SNPs may increase or decrease (Li et al., 2016). SDS and Tween 80 increased endothermic peak temperature and ΔH, but Span 80 lowered the ΔH of waxy corn SNPs, indicating that added surfactant provides a large steric hindrance to formation of hydrogen bonding between short-chain glucans. Consequently, it suppresses the formation of crystalline structure and causes the reduction of numerous crystals. On the other hand, small amounts of chitosan for monodisperse particles did not significantly affect the thermal behaviors of SNPs and showed similar thermal characteristics compared to native SNPs (Luo et al., 2019a).

Water content and storage temperature greatly influenced the retrogradation of SNPs. When the water content is relatively high (SNPs:water = 1:2 and 1:5), SNPs showed strong recrystallization tendency, and revealed relatively high ΔH (Steeneken and Woortman, 2009). Low temperature storage (4 °C) favored the formation of the double helix in SNPs compared to the storage at 25 °C (Gong et al., 2016). Regardless of the water content, ΔH increased with increasing storage time, indicating that retrogradation of SNPs was dependent on time.

Thermal properties of SNPs were depending on the hydrolyzate composition. In case of SNPs composed of only short-chain glucans, only one endothermic peak was observed at relatively high temperatures, however, two endothermic peaks were reported when total hydrolyzate was used (Demirkesen-Bicak et al., 2018). The low peak (Endotherm I) and high peak (Endotherm II) are considered gelatinization and aged amylose crystal melting, respectively. Therefore, the composition of the hydrolyzate may be a major factor affecting the thermal properties of the SNP.

## Molecular level studies using Fourier transform infrared spectroscopy (FTIR)

Structural changes of self-assembled SNPs have been studied using Fourier transform infrared spectroscopy (FTIR). The spectral change can be divided into band narrowing and change in absorption intensity of a particular band. Band narrowing indicates a decrease in the order and number of conformations, while changes in band strength reflect the long-range ordering and crystallinity (Sun et al., 2014b). As crystallization progresses, the arrangement of molecules is ordered, the range of conformation decreases, and the bond energy becomes smaller than the initial state, resulting in a narrower band (Wilson et al., 1991). In addition, the ratio of 1047 cm^−1^ and 1022 cm^−1^ reflects the ratio of the crystalline region to the amorphous region, which is an indicator of short-range ordered molecular structure (Mun and Shin, 2018; Smits et al., 1998; Warren et al., 2016).

In case of proso millet, the peak at 3465 cm^−1^ of native starch shifted to 3426 cm^−1^ in SNPs. Peak shifts to lower bands reflect increased intermolecular force of hydroxyl groups in the SNPs. In addition, the intensity of the peaks in the 2940–2970 cm^−1^ (C–H stretching) increased as the crystallization time was prolonged (Lian et al., 2013). Waxy corn SNPs with Span 80 showed a narrower peak in 3500–3400 cm^−1^ than native SNPs and SNPs with SDS and Tween 80. This can be attributed to the weak inter- and intra-molecular forces of the hydroxyl groups in the SNPs (Li et al., 2016). There was no significant difference among native SNPs, SDS-SNPs and Tween 80-SNPs in the ratio of 1045 cm^−1^/1022 cm^−1^, but that of SNPs with Span 80 was significantly reduced, indicating that short-range ordered structures are destroyed by Span 80 (Li et al., 2016).

Annealing and HMT treatments increased the spectral peak intensity, indicating a more perfect and ordered structure in agreement with XRD and thermal properties. The ratio of 1047 cm^−1^/1022 cm^−1^ in annealed SNPs was significantly higher than that of native SNPs and it increased with increasing annealing time. This indicated that annealing improved the crystallinity of SNPs (Ji et al., 2019). The increased ratios confirmed that the annealing system (high water content and thermal energy) allowed the transformation of double helix into crystal packing (Ji et al., 2019). HMT SNPs showed peaks in the 3700–3100 cm^−1^ range due to complex vibrational elongation and increased significantly when the SNPs are treated at 110 °C with a moisture content of 30% (Ji et al., 2015). Overall, annealing and HMT could increase the molecular rigidity of SNPs.

## Digestibility

Representative structural changes are those generated by a newly ordered crystalline structure via crystallization of linear short chain glucans debranched by pullulanase and isoamylase, which increases the content of RS and SDS (Kiatponglarp et al., 2015; Lee et al., 2010). Depending on the degree of debranching and the chain length of the hydrolyzate, the digestibility of starch can be varied (Liu et al., 2015; Shi et al., 2013; Suriya et al., 2018).

SNPs produced by self-assembly of supernatants are also a type of resistant starch, and several studies have reported on their digestibility (Liu et al., 2016; 2017). Liu et al. (2016) compared enzymatic hydrolysis kinetics of native corn starch, cooked corn starch, corn SNCs and corn SNPs. Corn SNPs showed the lowest hydrolysis rate and the slow hydrolysis rate of corn SNPs can be attributed to the compact structure of SNPs formed during recrystallization of short-chain glucans. In particular, the increase in the amount of short chains makes it more difficult for the enzyme to digest, unlikely corn SNCs which have pores and channels formed by acid hydrolysis resulting in relatively easy access of enzymes (Liu et al., 2017).

Digestibility of SNPs were changed by the type of crystal produced during self- assembly. In general, A-type crystal starch has lower digestibility than B-type crystal starch. However, SNPs obtained from short-chain glucans showed the opposite. The A-type crystal SNPs showed only 22.9% hydrolysis compared to 33.4% for the B-type crystal SNPs (Cai and Shi, 2014). This may be due to the structural feature difference between granular starch and particles as well as the differences in the number of crystalline packing patterns (monoclinic and hexagonal geometry) and water molecules (Cai and Shi, 2013; 2014). A-type crystal SNPs have a more compact structure with enzyme resistance, but B-type crystal SNPs are relatively less compact making easier enzyme penetration.

It is interesting to note that SNPs have been proposed as inhibitors of α-amylase (Jiang et al., 2018). As the concentration of SNPs increased, the inhibitory effect increased. SNPs changed the secondary structure of α-amylase resulting in inhibitory effect on α-amylase. This result suggests that SNPs have a potential as a α-amylase inhibitor and may be helpful in reducing the absorption of glucose for diabetics.

## Dispersibility

SNPs have less surface charge and tend to aggregate easily in an aqueous solution. This agglomeration makes it difficult to utilize SNPs in various fields, and therefore it is necessary to improve the dispersibility of SNPs. Dispersibility, uniform distribution of a solution, is an important factor for both biomaterials and biomedical applications and monodispersion is the most desirable (Ha et al., 2013).

To improve the stability of the dispersion, Liu et al. (2018) prepared SNPs (self-assembled using total hydrolysate) and modified it with sodium hypochlorite (NaClO) to induce a negative charge on the surface of the SNPs. As the degree of substitution increased, the negative charge increased, and the NaClO-SNPs dispersion (0.4%) substituted with 4 and 5% did not settle down even after 48 h. The settling rate was retarded due to the negative repulsive force generated by the carboxyl group. In contrast, Luo et al. (2019a) increased the positive charge on the surface of SNPs by adding chitosan, and the zeta potential rapidly increased in the positive direction. The SNPs positively charged on the surface by chitosan caused strong electrostatic repulsion between particle clusters, which inhibited undesirable aggregation and resulted in monodisperse particles. These results suggested that the cooperation of negative or positive functional groups can improve the dispersibility of SNPs.

The introduction of hydrophobic substituents also altered the surface properties of SNPs. When OSA-substituted SNPs are dispersed in different solutions (water, toluene, dichloromethane and chloroform), the highly substituted OSA-SNPs are more stable than native SNPs and less substituted OSA-SNPs possibly due to the reduction of interfacial tension (Jiang et al., 2016a). Addition of surfactants also improved dispersibility of SNPs (Li et al., 2016). Dispersion of waxy corn and potato SNPs with Tween 80 (HLB = 40) and SDS (HLB = 15) maintained their dispersibility up to 4 h. This is due to the increased affinity with water by the addition of surfactants with high HLB values. Depending on the dispersion characteristics of SNPs, not only agglomeration, but also colloidal creaming, coalescence, and sedimentation can be induced. As a factor affecting pipeline flow behavior, this may be an important consideration in industrial processing.

## Rheological properties

The viscosity and rheological properties of SNPs have been rarely reported. Jiang et al. (2016b) studied the rheological properties of SNC and SNP suspensions in sodium chloride solutions. The presence of ionic additives could affect the dispersibility of the particles and the rheological properties of the suspension. The apparent viscosity increased as content of both SNCs and SNPs increased, although viscosity of the SNPs was lower than that of the SNCs. The shear-thinning behavior of the SNP suspension was observed. SNPs suspension showed that viscosity behavior was dominant rather than gelling behavior. In many industries, including the food industry, rheological properties are important information for the processing of SNPs and are closely related to the physical properties of the final product, and further fundamental studies should be conducted.

Self-assembled SNPs are potentially valuable materials because they are environmentally friendly and do not require additional external energy. Because SNPs are natural materials, they can be a solution to resource depletion and environmental pollution compared to conventional plastic materials. Especially, in packaging, improvement of mechanical properties and low permeability by the addition of starch nanoparticles would be alternative of starch which have hydrophilic properties and poor mechanical properties. Furthermore, their applications are not only as RS or SDS, but also as a carrier and trapping agent of bioactive substances and drugs. The short linear α-d-(1,4) glucan with DP 20–35 produced by debranching of amylopectin is also known to be the best resources to produce RS type III, because of the formation and packing of double helix strands due to crystallization. These slow- or non-digestible fractions can be pioneered new commercial markets as healthy and functional ingredients. Additionally, SNPs can improve the bioavailability of bioactivity ingredients in human body by entrapping them as a nano-carrier.

Various factors such as starch concentration, enzyme type, debranching, and crystallization time affect the physicochemical properties of the final SNP. Therefore, the composition of the hydrolyzates we discussed in this review is also an important consideration. For example, SNPs with partial crystalline regions can be melted at low temperatures due to double helical structure of amylopectin under the heating process, affecting the texture of the food. If the hydrolysis composition and the characteristics of the SNPs obtained therefrom are modeled and standardized, various materials with controlled content of short-chain glucan and unbranched regions can be developed. Therefore, basic and fundamental research on SNPs is required and precise methods need to be developed.
